# Horticultural Therapy May Reduce Psychological and Physiological Stress in Adolescents with Anorexia Nervosa: A Pilot Study

**DOI:** 10.3390/nu14245198

**Published:** 2022-12-07

**Authors:** Olivia Curzio, Lucia Billeci, Vittorio Belmonti, Sara Colantonio, Lorenzo Cotrozzi, Carlotta Francesca De Pasquale, Maria Aurora Morales, Cristina Nali, Maria Antonietta Pascali, Francesca Venturi, Alessandro Tonacci, Nicola Zannoni, Sandra Maestro

**Affiliations:** 1 Institute of Clinical Physiology, National Research Council of Italy, Via Moruzzi 1, 56124 Pisa, Italy; 2 IRCCS Stella Maris Foundation, 56128 Pisa, Italy; 3 Institute of Information Science and Technologies “Alessandro Faedo” (ISTI), National Research Council of Italy, Via Moruzzi 1, 56124 Pisa, Italy; 4 Department of Agriculture, Food and Environment, University of Pisa, Via del Borghetto 80, 56124 Pisa, Italy; 5 Child and Adolescent Rehabilitation Clinic “Gli Orti di ADA”, Via dei Giacinti, 4, 56128 Pisa, Italy

**Keywords:** anorexia nervosa restricting type, eating disorders, green therapy, horticultural therapy, autonomic function, wearable sensors, wireless technologies, physiological monitoring, stress detection, olfactory stimuli

## Abstract

Studies in psychiatric populations have found a positive effect of Horticultural therapy (HCT) on reductions in stress levels. The main objective of the present pilot study was to evaluate the impact of the addition of HCT to conventional clinical treatment (Treatment as Usual, TaU) in a sample of six female adolescents with anorexia nervosa restricting type (AN-R), as compared to six AN-R patients, matched for sex and age, under TaU only. This is a prospective, non-profit, pilot study on patients with a previous diagnosis of AN-R and BMI < 16, recruited in 2020 in clinical settings. At enrolment (T0) and after treatment completion (TF), psychiatric assessment was performed. At T0, all the patients underwent: baseline electrocardiogram acquisition with a wearable chest strap for recording heart rate and its variability; skin conductance registration and thermal mapping of the individual’s face. An olfactory identification test was administered both to evaluate the olfactory sensoriality and to assess the induced stress. One-way analyses of variance (ANOVAs) were performed to analyze modifications in clinical and physiological variables, considering time (T0, TF) as a within-subjects factor and group (experimental vs. control) as between-subjects factors. When the ANOVA was significant, post hoc analysis was performed by Paired Sample T-tests. Only in the HCT group, stress response levels, as measured by the biological parameters, improved over time. The body uneasiness level and the affective problem measures displayed a significant improvement in the HCT subjects. HCT seems to have a positive influence on stress levels in AN-R.

## 1. Introduction

Eating disorders are burdened by a high rate of physical comorbidity, reaching, with anorexia nervosa (AN), the highest mortality rate of all mental disorders [1,2]. Thus, the need for multiple levels of treatment, including outpatient facilities as well as rehabilitation and hospital units, depends on the severity of the clinical picture. The onset of AN typically occurs during adolescence; the 15–19-year age group constitutes 40% of newly diagnosed cases; nearly 85% of cases have an onset within those of 20 years and, in almost all cases, symptoms start before the age of 25 years [3]. A lowering of the age of onset with an increase in new diagnoses at an early age (under 12 years) has been reported in the last few years [4,5,6].

Horticultural therapy and, more generally, Green therapy are considered natural complementary rehabilitation therapies, increasingly used in psychiatric pathologies in adult subjects [7]. Studies have also reported the contribution of such interventions on the reduction in stress levels and on the improvement in well-being of individuals at different ages, from healthy pediatric populations [8] to young adults [9], and even in elderly subjects [10,11]. Different studies have shown that the exposure to natural stimuli induces hyperactivity in the parasympathetic nervous system, ultimately resulting in a state of relaxation [12]. Exposure to nature implies a decrease in the autonomic arousal [13] and is beneficial to mood, through a decrease in negative emotions in favor of positive affective responses [14,15,16]. These responses to the exposure and the involvement in activities within natural environments occur in a few minutes and have a psycho-evolutionary value according to Ulrich. In other words, it would be an adaptive mechanism evolved in phylogeny to allow for rapid physical and emotional recovery after the recognition of a safe environment, especially after having faced an arousal phase. This rapid recovery, and the return to optimal operating conditions, would then be aimed at the quick reactivation of the necessary behaviors for survival, including the search for food and shelter. Studies in psychiatric [17] and non-psychiatric adult populations [9] have found a positive effect from Green and Horticultural therapy in reductions in stress levels. Such outcomes were evaluated through the variation in biological parameters, including cortisol [17], heart rate and its variability, measured by wearable sensors [9], as well as improvements in the subjective perception of stress [18]. However, there are no studies dealing with patients with Eating Disorders (EDs), specifically with AN, although the Green therapy setting should show several benefits to some specific vulnerabilities of these patients. In fact, while growing plants, the patients face the experience of taking care of other forms of life, getting them used to tolerate waiting times that are not always predictable: they have to deal with the imperfection of spontaneous growth of products in nature. Moreover, the horticultural products are characterized by specific and well-recognizable smells that may drive the selectivity of consumers toward food.

The main objective of the present pilot project was to evaluate the impact of Horticultural therapy, in addition to conventional clinical treatment (Treatment as Usual, TaU), in a sample of adolescents with anorexia nervosa restricting type (AN-R), as compared to a control group consisting of AN-R patients matched by sex, age, body mass index (BMI), duration of illness, and drug therapy, who carried out exclusively TaU. The standard multidisciplinary approach includes access to physical, nutritional, psychological, and psychiatric interventions.

The specific aims of the study were:

- To evaluate the impact of Horticultural Therapy (HCT) on the anorexic core psychopathology and psychiatric comorbidities;
- To assess the change in the stress level through the study of autonomic parameters (heart rate, heart rate variability, skin conductance, and facial thermography);
- To explore the olfactory function and its relationship with autonomic parameters as a possible variable of interest in response to odorous stimuli for both edible and non-edible compounds.

## 2. Materials and Methods

### 2.1. Study Design

The present study is a prospective, non-profit, pilot study that involved patients recruited in two settings, the clinical Unit for Child and Adolescence with EDs of the IRCCS Stella Maris and the Rehabilitation Unit for Child and Adolescents with EDs, “Gli Orti di Ada”.

The study aimed at evaluating the effects of a complementary treatment, the HCT, in patients with AN-R in addition to TaU.

### 2.2. Participants

At the time of enrolment, occurring in 2020, given the COVID-19-related pandemic period, the patients’ and families’ availability was checked before enrolling the AN-R patients. Parents and patients in the experimental group were provided with specific information on HCT and its benefits in other psychiatric pathologies. Then, each patient was randomly assigned to the experimental or to the control group. The sample was composed of 12 Caucasian female adolescents with AN-R (mean age: 14.86 ± 1.92 years; range: 11–18 years old): 6 in the experimental (TaU plus HCT) and 6 in the control (under TaU only) group. One subject had an illness duration of less than 2 years; 9 subjects had an illness duration between 1 and 2 years; 2 subjects had an illness duration of more than 2 years. At the time of enrollment and subsequent execution of the project, all the subjects were in a stable clinical condition and displayed good compliance with the treatment. The two groups were matched by sex, age, BMI, duration of illness, intensity of treatment, and drug therapy (*p* > 0.05).

Inclusion criteria: Diagnosis of AN-R; BMI > 16.

Exclusion criteria: Patients with severe AN-R in intensive hospital treatment, including enteral nutrition (BMI < 16); patients with severe psychiatric comorbidity.

The project received approval from the Pediatric Ethics committee for Tuscany Region (Florence, Italy; Register Number: 138/2020).

### 2.3. Interventions

#### 2.3.1. Treatment as Usual

In anorexia nervosa TaU is based on physical, nutritional, psychological, and psychiatric interventions. Multiple levels of this treatment are provided including hospitalization, outpatient treatments as well as rehabilitation units, according to the severity of the clinical symptoms. All the patients involved in this study underwent this kind of treatment that included nutritional counselling and rehabilitation, psychiatric, and psychopharmacological treatment as well as psychological intervention. All the patients underwent periodic clinical monitoring of biological parameters.

#### 2.3.2. Horticultural Therapy

Horticultural Therapy consisted of 24 sessions carried out twice a week for a total duration of 12 weeks (September–November 2020), with each session lasting around 45 min. These activities were performed in the garden and/or in a PVC tunnel greenhouse of the clinical unit, under the supervision and support of experts from the Department of Agriculture, Food and Environment (DAFE) of the University of Pisa and educators from the clinical units.

**Figure 1:**
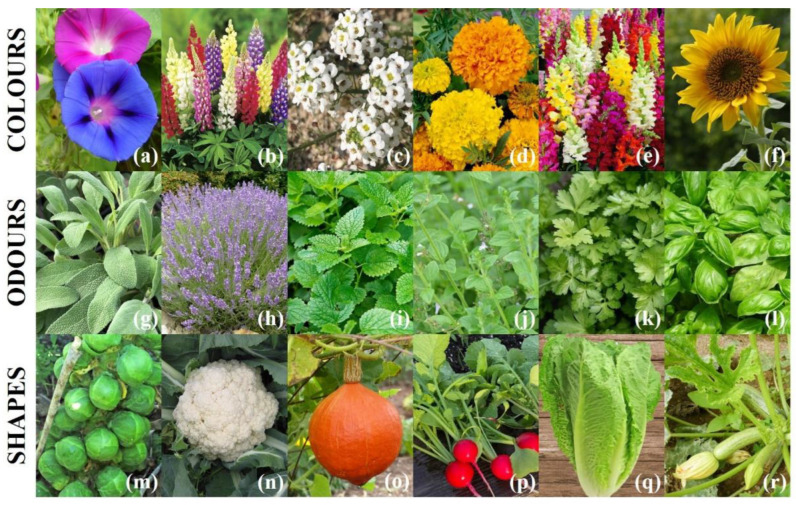
Plant species grown by patients in order to experience different sensory stimulations during the HTC. Color workstation: beach moonflower (**a**), lupin (**b**), madwort (**c**), marigold (**d**), snapdragon (**e**), sunflower (**f**). Odor workstation: common sage (**g**), lavender (**h**), lemon balm (**i**), lesser calamint (**j**), parsley (**k**), sweet basil (**l**). Shape workstation: Brussels sprouts (**m**), cauliflower (**n**), pumpkin (**o**), radish (**p**), romaine lettuce (**q**), zucchini (**r**).

In order to let the patient experience different sensory stimulations even without any specific sensorial training, the activities were organized in three workstations, each of which focused on six plant species selected to emphasize divergent colors, odors, and shapes (Figure 1). The colors group included (i) beach moonflower (*Ipomea violacea* L.), (ii) lupin (*Lupinus polyphyllus* L.), (iii) madwort (*Alyssum maritimum* L.), (iv) marigold (*Tagetes erecta* L.), (v) snapdragon (*Antirrhinum majus* L.), and (vi) sunflower (*Helianthus annuus* L.). The odors group included (i) common sage (*Salvia officinalis* L.), (ii) lavender (*Lavandula angustifolia* L.), (iii) lemon balm (*Melissa officinalis* L.), (iv) lesser calamint (*Calamintha nepeta* (L.) Savi), (v) parsley (*Petroselinum crispum* (Mill.) Fuss), and (vi) sweet basil (*Ocimum basilicum* L.). The shapes group included (i) Brussels sprouts (*Brassica oleracea* L. var. *gemmifera*), (ii) cauliflower (*Brassica oleracea* L. var. *botrytis*), (iii) pumpkin (*Cucurbita maxima* Duchesne), (iv) radish (*Raphanus sativus* L.), (v) romaine lettuce (*Lactuca sativa* var. l*ongifolia*), and (vi) zucchini (*Cucurbita pepo* L.). These species were also selected to include other variable plant characteristics such as the seed size and the growth timing and rate.

Patients from the experimental group were randomly sub-grouped in order to have two patients at each workstation. Sub-groups weekly alternated among the three workstations in order to let all the patients experience the different sensory stimulations.

At the beginning of the first session, DAFE experts (with the support of educators) introduced themselves and their work and provided the patients with some gadgets useful for HCT activities (e.g., backpacks, caps, notebooks, pens, and pencils). Then, they illustrated the HCT tools available at each workstation (e.g., seeds, pots, substrates, watering cans, fertilizers, tags, and sharpies) and explained the overall HCT design. A booklet prepared by DAFE experts and including major indications for growing the selected plant species (e.g., common and scientific names, pictures, and cultivation tips about the substrate to use, climatic needs, fertilizing, irrigation, and flowering) was also provided to each patient.

The next HCT sessions were organized as follows: (i) preparation for the session by educators, (ii) brief on-site explanation and fulfilment of the planned activities (e.g., substrate preparation, pot filling, sowing, labelling, watering, repotting, fertilizing, and taking care of plants) by DAFE experts, and (iii) observation and experiencing of developing plants.

**Figure 2:**
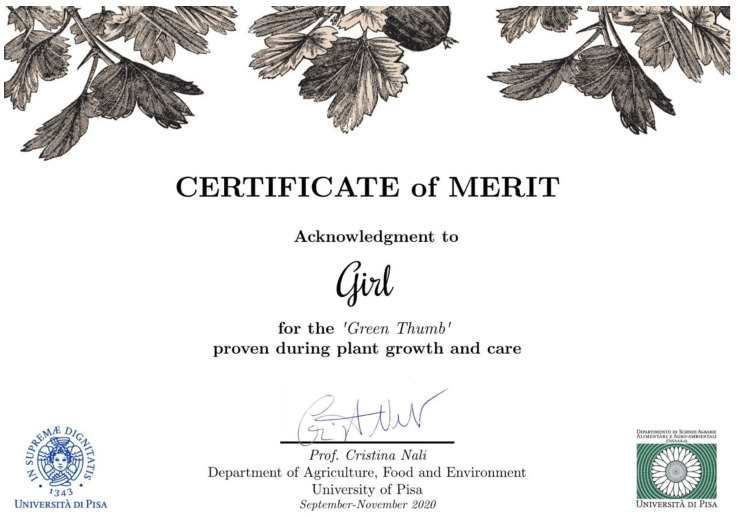
A ‘green thumb’ certification given to each patient in the experimental group at the end of the HCT.

At the end of the last session, a ‘green thumb’ certification, signed by a Full Professor of the DAFE and certifying the ability and commitment demonstrated in growing and taking care of plants, was given to each patient in the experimental group (Figure 2). An analogous certificate of merit, assessing the participation and commitment in the research activities, was given to each patient in the control group.

### 2.4. Procedure

During the whole testing procedure, patients were asked to sit comfortably in a chair and instructed to do nothing but relax.

At the enrolment, and after the treatment completion, within completely dedicated sessions, psychiatric assessment was performed by the administration of the following standardized instruments: Eating Disorder Inventory-3 (EDI-3) [19]; Body Uneasiness Test (BUT) [20]; and Children Depression Inventory (CDI) [21].

At T0, i.e., before the beginning of the HCT sessions, all the patients underwent a baseline acquisition of: (i) electrocardiogram (ECG) with a wearable chest strap; (ii) skin conductance (SC) with a wearable sensor placed on two adjacent fingers of the non-dominant hand; (iii) thermal mapping of the individual’s face with thermal imaging camera. The total duration of the acquisition was about 10 min.

Given that, among sensorial features, the smell profile of a food seems to have the main influence on consumers’ food choice, an olfactory identification test was also administered to evaluate the olfactory sensoriality relating to 12 smells (6 of them related to edible compounds and the other 6 related to non-edible ones), some of which were referring to the world of the garden. After a short introduction, the patients had to try to identify the olfactory compound contained within the solution developed ad hoc by researchers from DAFE of the University of Pisa. During the test, the response of the autonomic nervous system to olfactory stimuli was measured through the analysis of heart rate (HR) and heart rate variability (HRV), derived from the ECG and SC, as previously described [22,23]. A thermal video sequence (including subject acclimatization, olfactory stimuli, and subject relaxation) was also acquired to investigate the temperature changes in facial landmarks (e.g., nose tip, nasal septum, forehead) possibly related to physical or mental stress [24].

The Green and Ortho-Therapy sessions, according to the protocol described above, lasted 12 weeks, with 2 sessions per week, for a total of 24 sessions.

After the conclusion of the Green and Ortho-Therapy, a complete assessment of the stress-dependent autonomic parameters in resting condition (HR, HRV, SC, and facial thermal imaging) was repeated in all 12 subjects. Similarly, the olfactory identification test and the administration of psychopathological assessment by EDI-3, BUT, and CDI were repeated in all subjects in both groups.

### 2.5. Instruments

#### 2.5.1. Model Solutions Used for the Olfactory Stimulation

In Table 1, the odorous solutions used for the olfactory stimulation are reported. To reduce bias derived from the repetition of the olfactory test, two subsets of odors slightly different were used during the two olfactory trials (i.e., at T0 and TF).

#### 2.5.2. ECG Acquisition and Processing

The acquisition of the ECG signal was performed using a commercial wearable, Bluetooth-equipped single-lead sensor, named Shimmer ECG (Shimmer Sensing, Dublin, Republic of Ireland), attached to a commercial fitness-like chest strap (Polar Electro Oy, Kempele, Finland) with a sampling frequency of 500 Hz. This device was already tested in AN in our previous studies [25,26].

The ECG signal was analyzed using a dedicated routine implemented in MATLAB (The MathWorks, Inc., Natick, MA, USA). ECG signals were pre-processed for artifact removal, QRS complexes were detected [27], and then the RR series were reconstructed and corrected. The correction was applied to remove non-sinusoidal beats in order to obtain an RR series that only contains variations due to the sinus node, thus, reflecting the activity of the ANS [28].

From the corrected RR series, a number of significant features was extracted [29]:

Time-domain features:
  o Heart rate (HR): number of heart pulses per unit of time. Measured in beats per minute (bpm);
  o Standard deviation of the normal R–R intervals (SDNN): measured in ms, it is an estimate of the HRV influenced by both the sympathetic and para-sympathetic branches of the ANS;
  o Root mean square of the successive differences (RMSSD): measured in ms, it represents the root mean square of the differences between neighboring R–R intervals. It is an estimate of the parasympathetic activity of the ANS;
  o Number of normal R–R intervals differing for more than 50 ms (NN50): it estimates the number (or the percentage) of the normal R–R intervals differing for more than 50 ms from each other. Under resting state short-term recordings, it refers to the parasympathetic activity of the ANS.
Frequency-domain features:
  o Low frequency (LF): power spectral density of the ECG signal at low frequencies (0.04–0.15 Hz), employed as an estimator of the sympathetic activity of the ANS;
  o High frequency (HF): power spectral density of the ECG signal at high frequencies (0.15–0.4 Hz), employed as an estimator of the sympathetic and parasympathetic activity of the ANS;
  o Low- to high-frequency component ratio (LF/HF): it indicates the overall balance between low- and high-frequency components of the ECG signal.

#### 2.5.3. GSR Acquisition and Processing

The acquisition of the GSR signal was conducted with a commercial wearable sensor, Shimmer3GSR (Shimmer Sensing, Dublin, Republic of Ireland), communicating via Bluetooth to the manufacturer user interface, with a sampling frequency of 51.2 Hz. The GSR sensor captured the corresponding signal, being attached to two adjacent fingers of the subject’s non-dominant hand at the phalanx level with the support of two comfortable soft rings in turn worn by the individual studied. This device was also previously tested by our group in AN subjects [30].

Concerning the processing, the GSR signal was analyzed using the Matlab-based tool Ledalab [31]. The GSR signal was initially filtered with a first-order Butterworth low-pass filter at 5 Hz to remove high-frequency noise, and then continuous decomposition analysis was applied for the extraction of both tonic and phasic activities. As such, the following features were extracted:

o Tonic GSR component: mainly refers to slow changes in the electrical skin signal, dominant at rest and during relaxing activities, not including specific stimuli;
o Phasic GSR component: extracted to study the response to the sensory (olfactory) stimulation, as it refers to quick responses to specific stimuli (also known as Skin Conductance Response, SCR).

#### 2.5.4. Thermal Imaging Acquisition and Processing

Thermography is a contactless measure that reflects subcutaneous blood distribution and sweat secretion. In the literature, stress elicited by mental tests was observed to induce an enhancement in blood flow in the forehead, supraorbital, and frontal vessels [32]. The mean temperature of the face could hardly be considered as a reliable stress indicator; so far, the most reliable facial feature to identify stress arising is the nose tip, which has been successfully exploited to design and develop a real-time classification algorithm to improve social robots’ interaction [33,34]. In a more recent work, the nose tip and the nasal septum were also established as stable stress indicators [24]. The only work studying the relation between AN and thermal measurements was published in 2016 and aimed at detecting the disturbance in thermoregulation mechanisms and body temperature of AN subjects [35] being more focused on providing clinical implications for the treatment of anorexic patients.

Hence, in our experimentation, we decided to focus on small circular Regions of Interest (ROIs) around the facial landmarks, which were found in the literature to be most likely related to stress: the nose tip, the nasal septum, and the forehead (left and right). The thermal signal was measured using an FLIR A65sc infrared camera with a focal length of 13 mm (spectral range of 7.5–13 µm (LWIR), resolution of 640 × 512 pixels, thermal sensitivity <0.05 °C, and streaming rate 7.5 Hz).

For each subject a long video sequence of about 10 min was acquired at T0 and at TF. The acquisition includes three main phases: the acclimatization of the subject in the room, the sequence of the olfactory stimuli, and the recovery (or resting phase). The thermal signal was extracted manually as the mean temperature of each ROI at three specific time steps: during the acclimatization, in the middle of the olfactory test, and at the end of the test, while relaxing. Thermal features were extracted from the nose tip, the nasal septum, the right forehead, the left forehead, and the mean of the two forehead ROIs normalized with respect to the nose tip temperature (the difference between the average of the two forehead ROIs and the nose tip). The statistical analysis was carried out only for the measurements acquired during acclimatization and resting phase, in order to observe how fast the recovery is from the stress status induced by the olfactory test.

### 2.6. Endpoint of the Study

The main endpoint of the study was to evaluate T0-TF differences (before and after the Green and Horticultural Therapy sessions), both in the experimental group and in the control group, of the following measures:

(1) Psychometric questionnaires, clinical variables: Eating Disorder Inventory-3 (EDI-3) [19]; Body Uneasiness Test (BUT) [20]; and Children Depression Inventory (CDI) [21];
(2) Basal level of stress evaluated by autonomic measures (values of Skin Conductance, SC; Heart Rate, HR and its variability, HRV; and facial thermal imaging);
(3) Olfactory identification test (the number of correctly identified stimuli);
(4) Level of stress during the olfactory test evaluated by autonomic response (values of Skin Conductance, SC, Heart Rate, HR, and its variability, HRV, and thermal mapping of the face, in response to olfactory stimuli).

### 2.7. Statistical Analysis of the Results

The normality of the variables was determined using the Shapiro–Wilk normality test. Since the variables were normally distributed, one-way analyses of variance (ANOVAs) were used to analyze variables considering time (i.e., T0, TF) as within-subjects factor and group (experimental vs. control) as between-subjects factor. When the ANOVA was significant, post hoc analysis was performed by Paired Sample T-tests. A value of *p* < 0.05 was considered as significant.

## 3. Results

### 3.1. Olfactory Stimuli

The number of correctly identified stimuli did not change over time (T0: control: 3.80 ± 1.85, experimental: 5.10 ± 1.08; TF: control: 4.20 ± 0.97, experimental: 5.30 ± 1.03) and it was not different between groups. This experimental evidence can be well explained by the fact that recruited subjects belonging to both the two groups were not involved in training specifically aimed at improving olfactory identification abilities in the time frame between the first (T0) and the last (TF) sessions.

### 3.2. Clinical Assessment: Core Psychopathology and Psychiatric Comorbidities Variation

Between T0 and TF, the experimental and the control groups showed significant differences in some dimensions of the questionnaires administered (Table 2). In particular, the BUT and the subscales Impulse to Thinness and Affective Problem of the EDI displayed a significant improvement in the experimental group and a significant worsening in the control group. The CDI displayed a significant improvement in both the two groups (see Table 2).

### 3.3. Cardiac Variables

Results regarding the cardiac variables are reported in Table 3.

In the baseline condition, LF/HF decreased at TF compared to T0, specifically, in the experimental group. Since this index expresses the sympathetic/parasympathetic balance, a decrease at TF may indicate that the subjects are less stressed in the phase of preparation to the execution of the odor identification task compared to T0. This is more evident in the subjects who performed HCT, even without any improvement in their olfactory perception. Thus, this result suggests a possible reduction in the stress condition of the subjects during resting.

During olfactory stimulation (Task ON), the LFn decreased in both groups, with a slightly more marked decrease in the experimental group, coherently with the assumption above. This is in line with current literature, which indicates a suppression of the sympathetic stimuli due to olfactory stimulation.

The results during the inter-stimulus stimulation (Task OFF) are a bit more contradictory since we observed an increase in NN50, mainly related to the parasympathetic activity, but also an increase in LFn, mainly related to sympathetic activity, in particular in the experimental group. These results could indicate a realignment of the ANS during the inter-stimulus interval, with a more pronounced activation of the sympathetic system in the experimental group (LFn), whereas the contradictory result of NN50 could be even due to the small sample size analyzed.

### 3.4. Skin Conductance Responses

As for the GSR signal, a significant difference in Non-Specific Skin Conductance Responses (NSCRs) during the Task OFF between T0 and TF only in the experimental group was reported, suggesting that the experimental-group individuals had a higher tendency towards relaxation just after the end of each odor presentation compared to the control group, possibly due to the HCT protocol (Table 4).

### 3.5. Thermal Variables

As for the thermal imaging, Table 5 shows a list of the temperature features acquired from the facial ROIs considered most relevant to assess the stress status induced by the olfactory test. Table 5 reports the mean thermal variables at T0 and TF, both during the acclimatization and the resting phase, for control and experimental groups. It is worth mentioning that for all the thermal measurements (regarding both the time effect and the time effect per group), the statistical significance was assessed by computing the *p*-values. This meant that when considering the time effect, the p is always less than 0.01, while when considering the time effect per group, the p is always greater than 0.1.

Columns T0 and TF in Table 6 show the differences in temperature between the acclimatization and resting phase, at T0 and at TF, grouped with respect to the subject group (control and experimental) and to the specific ROI measured. In the last column in Table 6, it was remarked for which ROI the temperature variation between resting and acclimatization is reduced at TF (with respect to T0), as a cue of a better stress recovery, enhanced in the experimental group.

The analysis shows that the absolute value of the difference between the resting and the acclimatization phase decreases for all the ‘thermal markers’ but one (i.e., the frontal left), suggesting a generalized better recovery from the stress induced by the olfactory stimuli for the experimental group than for the control group.

Hence, even if we cannot consider this finding statistically sound, mainly due to the size of the population study, we believe that the results presented do justify further efforts in planning a larger experimentation, exploiting facial thermal markers to assess both the response to stress stimuli and, therefore, the effectiveness of HCT for the AN subject.

## 4. Discussion

The results presented in the previous section consistently point out that the HCT setting may positively affect the psychological status of the AN-R subjects enrolled in this pilot study, while reducing the stress induced by an olfactory test, specifically designed. The stress level was assessed at baseline, at the beginning and at the end of the experiment, through standard and experimental methodologies for physiological monitoring (HR, HRV, SC, facial thermal imaging). The statistical analysis carried out for all the acquired signals shows that for the clinical variables as well for HRV and SC, it is not possible to draw robust conclusions, due to the low significance of the differences between groups (experimental and control) from T0 to TF. On the contrary, the thermal variables seem to be more robust and statistically relevant.

Disregarding the statistical significance for now, the present study provides a preliminary confirmation of the benefits of HCT, both in reducing stress levels and in alleviating some specific psychological vulnerabilities of eating disorders. In fact, in the experimental group, the stress response level, as measured by various biological parameters, improved over time in comparison to the group of subjects who did not undergo HCT. In the subjects who participated in the HCT sessions, we observed an improvement in some psychological measures, i.e., the drive for thinness, body dissatisfaction, inadequacy, interpersonal insecurity, and affective problems. It is interesting to note that these dimensions represent the core constellation of the maintaining factor of EDs. Notably, the growth rhythms of the crops require patience, waiting, tolerance, and are beyond the control of the subject. In this sense, the HCT setting represents a good context for the development and recovery of emotional attitudes compromised in this disease condition. Obviously, the tutors, both the educators and the agronomist experts, had a fundamental role in mediating the experience, contributing with the transmission of knowledge and emotional reinforcement, so as to motivate the AN-R subjects.

Although throughout the HCT sessions, the subjects were exposed with greater intensity and regularity to the scents and odors of plants, without any specific training for olfactory assessment, the ability to discriminate odors did not show any improvement from T0 to TF, regardless of the group we consider.

However, at TF, the level of stress response to olfactory stimuli was decreased only in the subjects who underwent HCT, while this improvement was not observed in the control group. The reduction in the stress associated with the sensory experience is an encouragement to continue the research on this topic. In a future perspective, the effect of a specific olfactory training, arranged according to what was previously described by Tonacci and colleagues [23], in patients with AN, should also be explored, even in combination with HCT.

Indeed, the olfactory function has an important relationship with the area of emotional memory and may be involved in other psychiatric impairments. In our previous study, we observed that in AN patients, although the ability to discriminate olfactorily is not impaired overall, it is correlated with the autistic component [36]. It is also important to consider the effects of undernutrition on encephalic structures and neuroendocrine modulation, which affects many psychic functions [37]. On the other hand, improvement in this function could be an important therapeutic target given the central role of this sensory channel.

## 5. Conclusions

In this pilot study, we presented a preliminary assessment of the effect of horticultural therapy in adolescents with anorexia nervosa. To the best of our knowledge, this is the first study in which such treatment was experimented with in patients with eating disorders.

Despite the low sample size, the results of our study suggested that the group of patients who underwent the horticultural therapy showed a clinical improvement in the level of bodily distress and the measure of affective problems. Moreover, in this group of subjects, we also observed a reduction in the level of stress at resting as well as a reduction in the stressful response to olfactory stimuli, as measured by physiological parameters over time. Hence, we would like to remark that there is some evidence concurring in the direction of a positive assessment of the impact of HCT, in addition to conventional clinical treatment, for individuals with a diagnosis of AN-R. The experimentation protocol seems to be convincing as it is and possibly reproducible at a larger scale, to improve statistical robustness. In conclusion, Green therapy experiences and specifically HCT should be proposed as useful activities for reducing stress levels in adolescents with food-related issues diagnosed with AN-R. Reducing stress levels is related to improving clinical parameters and can go a long way toward affecting the subject’s psychopathology, particularly by going to work on the behavioral, cognitive, and emotional rigidities associated with the tendency toward food restriction and the more general psychopathological picture of these young patients.

For future research, we plan to initiate and carry out trials, in order to ensure a greater statistical robustness by increasing the size of the study population; in this perspective, a careful statistical power analysis is required in order to justify the sample size [38], taking into account that large sample size could be a challenge in the case of subjects with AN, who are often difficult to recruit [39].

## Figures and Tables

**Table 1:** Odorous solutions used for the stimulation. Solutions were stored for 24 h in the dark at room temperature (20 ± 1 °C).

Sample Code	Odour Family	Specific Descriptor	Recipe
First trial			
1	Fruity	Apricot	80 mL of commercial juice + 100 mL of white table wine
2	Fresh vegetal	Tomato	80 mL of commercial juice + 100 mL of white table wine
3	Fresh vegetal	Asparagus	60 mL of cooking water + 100 mL of white table wine
4	Balsamic/woody	Swiss Pine	Commercial essential oil diluted in white table wine (1% *v/v*)
5	Fruity	Orange	80 mL of commercial juice + 100 mL of white table wine
6	Floral	Lavender	Commercial essential oil diluted in white table wine (1% *v/v*)
7	Mediterranean spicy	Basil	Commercial essential oil diluted in white table wine (1% *v/v*)
8	Fresh vegetal	Olive oil	Pure EVOO
9	Fruity	Grapefruit	80 mL of commercial juice + 100 mL of white table wine
10	Balsamic	Mint	Commercial essential oil diluted in white table wine (1% *v/v*)
11	Earthy	Dirt	Pure topsoil
12	Mediterranean spicy	Thyme	Commercial essential oil diluted in white table wine (1% *v/v*)
Second trial			
1	Fruity	Peach	80 mL of commercial juice + 100 mL of white table wine
2	Fresh vegetal	Tomato	80 mL of commercial juice + 100 mL of white table wine
3	Fresh vegetal	Sage	Commercial essential oil diluted in white table wine (1% *v/v*)
4	Balsamic/woody	Swiss Pine	Commercial essential oil diluted in white table wine (1% *v/v*)
5	Fruity	Orange	80 mL of commercial juice + 100 mL of white table wine
6	Floral	Lavender	Commercial essential oil diluted in white table wine (1% *v/v*)
7	Mediterranean spicy	Basil	Commercial essential oil diluted in white table wine (1% *v/v*)
8	Fresh vegetal	Olive oil	Pure EVOO
9	Fruity	Tangerine	80 mL of commercial juice + 100 mL of white table wine
10	Balsamic	Peppermint	Commercial essential oil diluted in white table wine (1% *v/v*)
11	Resinous	Scots pine	Commercial essential oil diluted in white table wine (1% *v/v*)
12	Mediterranean spicy	Thyme	Commercial essential oil diluted in white table wine (1% *v/v*)

**Table 2:** Difference in clinical variables from T0 to TF in control and experimental groups. Variables are expressed as Mean (SD). * *p* < 0.05, ** *p* < 0.01.

Variable	T0	TF	Time Effect	Time × Group Effect	Post-Hoc
Body Mass Index			*p* = 0.17	*p* = 0.96	
Control	16.56 (1.18)	17.12 (0.86)			
Experimental	17.33 (1.31)	17.92 (1.23)			
Child Depression Inventory			*p* = 0.04 *	*p* = 0.47	
Control	42.50 (29.08)	22.50 (3.42)			*p* = 0.25
Experimental	30.00 (8.12)	19.60 (12.28)			*p* = 0.02 *
Body Uneasiness Test			*p* = 0.96	*p* = 0.005 **	
Control	2.18 (1.27)	3.39 (1.71)			*p* = 0.02 *
Experimental	3.40 (0.74)	2.23 (1.49)			*p* = 0.08
Eating Disorder Inventory					
Impulse to thinness			*p* = 0.46	*p* = 0.04 *	
Control	17.60 (8.82)	21.40 (10.41)			*p* = 0.02 *
Experimental	23.80 (4.55)	16.20 (8.89)			*p* = 0.08
Bulimia			*p* = 0.70	*p* = 0.45	
Control	4.40 (3.67)	5.60 (5.86)			
Experimental	2.80 (2.17)	2.40 (3.91)			
Dissatisfaction			*p* = 0.37	*p* = 0.18	
Control	24.60 (14.08)	26.60 (15.29)			
Experimental	29.20 (9.06)	19.80 (15.07)			
Risk			*p* = 0.47	*p* = 0.11	
Control	46.60 (25.09)	53.60 (29.16)			
Experimental	55.80 (13.77)	38.40 (29.50)			
Ineffectiveness			*p* = 0.28	*p* = 0.08	
Control	25.60 (17.30)	29.20 (17.08)			
Experimental	36.80 (8.95)	23.20 (14.82)			
Interpersonal Problems			*p* = 0.05	*p* = 0.15	
Control	22.80 (11.77)	21.00 (10.29)			
Experimental	29.80 (6.38)	19.20 (12.26)			
Affective Problems			*p* = 0.53	*p* = 0.03 *	
Control	23.60 (16.56)	34.00 (20.83)			*p* = 0.17
Experimental	38.25 (7.54)	21.25 (17.84)			*p* = 0.03 *
Overcontrol			*p* = 0.72	*p* = 0.15	
Control	21.60 (10.94)	28.40 (16.30)			
Experimental	25.20 (10.13)	21.00 (13.07)			

**Table 3:** Difference in cardiac variables from T0 to TF in control and experimental groups. Variables are expressed as Mean (SD). * *p* < 0.05, ** *p* < 0.01.

Variable	T0	TF	Time Effect	Time × Group Effect	Post-Hoc
Baseline					
HR			*p* = 0.35	*p* = 0.21	
Control	69.41 (21.01)	71.07 (6.17)			
Experimental	80.32 (10.56)	69.84 (13.24)			
SDNN			*p* = 0.21	*p* = 0.86	
Control	0.04 (0.15)	0.05 (0.02)			
Experimental	0.04 (0.19)	0.06 (0.02)			
RMSSD			*p* = 0.23	*p* = 1.00	
Control	0.05 (0.02)	0.06 (0.02)			
Experimental	0.04 (0.03)	0.05 (0.03)			
NN50			*p* = 0.19	*p* = 0.47	
Control	25.00 (6.83)	32.43 (7.05)			
Experimental	19.00 (21.04)	21.33 (17.57)			
LFn			*p* = 0.27	*p* = 0.29	
Control	0.17 (0.04)	0.17 (0.04)			
Experimental	0.29 (0.13)	0.23 (0.12)			
HFn			*p* = 0.38	*p* = 0.14	
Control	0.96 (0.79)	0.85 (0.35)			
Experimental	0.51 (0.48)	0.92 (0.69)			
LF/HF			*p* = 0.03 *	*p* = 0.35	
Control	0.62 (0.79)	0.26 (0.14)			*p* = 0.31
Experimental	1.64 (1.53)	0.86 (1.08)			*p* = 0.04 *
Task ON					
HR			*p* = 0.82	*p* = 0.59	
Control	73.73 (22.21)	75.34 (5.33)			
Experimental	82.61 (10.35)	78.74 (14.44)			
SDNN			*p* = 0.15	*p* = 0.49	
Control	0.05 (0.02)	0.05 (0.02)			
Experimental	0.07 (0.03)	0.06 (0.02)			
RMSSD			*p* = 0.28	*p* = 0.80	
Control	0.05 (0.03)	0.06 (0.02)			
Experimental	0.03 (0.02)	0.04 (0.02)			
NN50			*p* = 0.02 *	*p* = 0.54	
Control	10.23 (2.65)	4.87 (1.38)			*p* = 0.05
Experimental	6.93 (7.15)	3.46 (2.32)			*p* = 0.20
LFn			*p* < 0.001 **	*p* = 0.25	
Control	0.21 (0.04)	0.11 (0.01)			*p* = 0.007 **
Experimental	0.25 (0.05)	0.11 (0.01)			*p* = 0.002 **
HFn			*p* = 0.15	*p* = 0.09	
Control	0.65 (0.60)	0.23 (0.13)			
Experimental	0.21 (0.17)	0.25 (0.19)			
LF/HF			*p* = 0.05	*p* = 0.22	
Control	1.49 (1.60)	0.94 (0.62)			
Experimental	3.18 (2.44)	1.06 (0.88)			
Task OFF					
HR			*p* = 0.45	*p* = 0.47	
Control	74.50 (21.01)	74.40 (6.81)			
Experimental	85.29 (9.06)	78.23 (14.60)			
SDNN			*p* = 0.69	*p* = 0.26	
Control	0.08 (0.03)	0.05 (0.02)			
Experimental	0.06 (0.02)	0.06 (0.02)			
RMSSD			*p* = 0.47	*p* = 0.41	
Control	0.06 (0.03)	0.05 (0.02)			
Experimental	0.04 (0.02)	0.04 (0.02)			
NN50			*p* = 0.001 **	*p* = 0.35	
Control	5.14 (0.75)	13.13 (2.68)			*p* = 0.01 *
Experimental	2.78 (2.07)	8.32 (5.33)			*p* = 0.03 *
LFn			*p* = 0.001 *	*p* = 0.17	
Control	0.11 (0.03)	0.18 (0.05)			*p* = 0.11
Experimental	0.10 (0.03)	0.23 (0.04)			*p* = 0.004 **
HFn			*p* = 0.12	*p* = 0.38	
Control	0.37 (0.36)	0.44 (0.21)			
Experimental	0.15 (0.07)	0.40 (0.34)			
LF/HF			*p* = 0.46	*p* = 0.46	
Control	0.78 (0.60)	0.79 (0.43)			
Experimental	1.39 (1.00)	2.16 (2.58)			
Recovery					
HR			*p* = 0.96	*p* = 0.40	
Control	66.89 (17.77)	70.81 (2.52)			
Experimental	78.95 (9.34)	74.61 (14.81)			
SDNN			*p* = 0.25	*p* = 0.48	
Control	0.05 (0.02)	0.05 (0.02)			
Experimental	0.04 (0.01)	0.05 (0.02)			
RMSSD			*p* = 0.68	*p* = 0.29	
Control	0.05 (0.02)	0.05 (0.01)			
Experimental	0.03 (0.02)	0.04 (0.02)			
NN50			*p* = 0.78	*p* = 0.07	
Control	31.91 (5.39)	26.50 (8.02)			
Experimental	19.54 (18.39)	23.66 (16.15)			
LFn			*p* = 0.81	*p* = 0.83	
Control	0.26 (0.12)	0.24 (0.12)			
Experimental	0.27 (0.12)	0.27 (0.14)			
HFn			*p* = 0.74	*p* = 0.13	
Control	1.03 (0.72)	0.78 (0.27)			
Experimental	0.44 (0.34)	0.82 (0.67)			
LF/HF			*p* = 0.45	*p* = 0.44	
Control	0.59 (0.77)	0.58 (0.61)			
Experimental	1.42 (1.25)	3.54 (6.56)			

**Table 4:** Difference in skin conductance variables from T0 to TF in control and experimental groups. Variables are expressed as Mean (SD). * *p* < 0.05.

Variable	T0	TF	Time Effect	Time x Group Effect	Post-Hoc
Baseline					
Tonic			*p* = 0.09	*p* = 0.21	
Control	1.31 (0.94)	1.07 (0.60)			
Experimental	2.16 (1.22)	0.81 (0.44)			
Task ON					
Tonic			*p* = 0.05	*p* = 0.47	
Control	2.74 (2.30)	1.29 (0.87)			
Experimental	4.16 (1.94)	1.42 (1.33)			
Phasic			*p* = 0.15	*p* = 0.70	
Control	0.24 (0.24)	0.04 (0.02)			
Experimental	0.51 (0.50)	0.17 (0.34)			
nSCR			*p* = 0.05	*p* = 0.89	
Control	6.33 (2.82)	2.82 (1.98)			
Experimental	6.83 (1.93)	3.88 (3.24)			
Task OFF					
Tonic			*p* = 0.05	*p* = 0.41	
Control	2.73 (2.21)	1.30 (0.86)			
Experimental	4.38 (2.11)	1.42 (1.31)			
Phasic			*p* = 0.06	*p* = 0.34	
Control	0.14 (0.31)	0.06 (0.05)			
Experimental	0.30 (0.21)	0.08 (0.12)			
nSCR			*p* = 0.01 *	*p* = 0.10	
Control	14.94 (7.31)	11.19 (5.47)			*p* = 0.41
Experimental	23.78 (4.33)	10.71 (4.52)			*p* = 0.01 *
Recovery					
Tonic			*p* = 0.06	*p* = 0.32	
Control	1.80 (0.89)	0.92 (0.55)			
Experimental	3.87 (1.83)	1.42 (1.14)			

**Table 5:** Thermal variables from T0 to TF in control and experimental groups. Variables are expressed as Mean (SD). * *p* < 0.05, ** *p* < 0.01.

Variable	T0	TF	Time Effect	Time × Group Effect	Post-Hoc
Acclimatization					
Nose			*p* = 0.01 *	*p* = 0.94	
Control	31.38 (5.68)	26.14 (2.08)			*p* = 0.10
Experimental	35.15 (1.63)	30.15 (4.49)			*p* = 0.09
Septum			*p* = 0.04 *	*p* = 0.875	
Control	33.36 (3.55)	31.37 (2.21)			*p* = 0.26
Experimental	35.03 (1.46)	32.40 (2.15)			*p* = 0.09
Frontal right			*p* = 0.005 **	*p* = 0.61	
Control	35.80 (1.75)	34.39 (1.03)			*p* = 0.14
Experimental	36.14 (0.39)	34.27 (0.66)			*p* = 0.006 **
Frontal left			*p* = 0.04 *	*p* = 0.31	
Control	35.47 (2.06)	34.79 (0.57)			*p* = 0.52
Experimental	36.23 (0.39)	34.43 (0.55)			*p* = 0.005 **
Nose-frontal §			*p* = 0.03 *	*p* = 0.77	
Control	4.25 (3.98)	8.16 (1.15)			*p* = 0.10
Experimental	1.03 (1.30)	4.11 (4.26)			*p* = 0.22
Resting					
Nose			*p* = 0.005 **	*p* = 0.78	
Control	33.71 (5.21)	27.45 (3.53)			*p* = 0.07
Experimental	35.60 (2.09)	30.22 (3.51)			*p* = 0.02 *
Septum			*p* = 0.01 *	*p* = 0.81	
Control	34.43 (3.12)	31.66 (2.38)			*p* = 0.18
Experimental	35.71 (1.23)	32.48 (1.95)			*p* = 0.01 *
Frontal right			*p* < 0.001 **	*p* = 0.94	
Control	36.42 (1.45)	34.21 (1.28)			*p* = 0.02 *
Experimental	36.41 (0.41)	34.25 (0.70)			*p* = 0.003 **
Frontal left			*p* = 0.001 **	*p* = 0.35	
Control	36.12 (1.70)	34.62 (0.60)			*p* = 0.09
Experimental	36.55 (0.79)	34.28 (0.60)			*p* = 0.005 **
Nose-frontal §			*p* = 0.01 *	*p* = 0.58	
Control	2.56 (3.97)	7.25 (3.41)			*p* = 0.07
Experimental	0.87 (1.84)	4.13 (3.45)			*p* = 0.11

§ Nasal-frontal = the difference between: the mean between the two ROIs of the forehead and the nose tip.

**Table 6:** Difference in facial ROI from T0 to TF in control and experimental groups, with a focus on the reduction in temperature variation between acclimatization and resting phase at TF with respect to TF.

Facial ROI	Subject Group	T0	TF	Temp. Variation (%)	Temp. Variation
					Reduced
Nose	Control	2.33	−1.31	43.78	TRUE
	Experimental	0.45	0.07	84.44	
Septum	Control	1.07	0.29	72.90	TRUE
	Experimental	0.68	0.08	88.23	
Frontal right	Control	0.62	−0.18	70.97	TRUE
	Experimental	0.27	−0.02	92.59	
Frontal left	Control	−1.35	−0.17	87.41	FALSE
	Experimental	0.32	−0.15	53.13	
Nose-Frontal	Control	−1.69	−0.91	46.15	TRUE
	Experimental	−0.16	0.02	87.5	

## Data Availability

The data presented in this study are available on request from the corresponding author.
